# A one‐pot process for synthesis of mitomycin analogs catalyzed by laccase/lipase optimized by response surface methodology

**DOI:** 10.1002/elsc.201900118

**Published:** 2019-09-30

**Authors:** Yuanyuan Zhang, Quancai Yao, Zewen Li, Fengke Yang, Fanye Wang, Junhong Liu

**Affiliations:** ^1^ Department of Pharmaceutical Engineering in College of Chemical Engineering Qingdao University of Science and Technology Qingdao P. R. China; ^2^ State Key Laboratory Base for Eco‐Chemical Engineering in College of Chemical Engineering Qingdao University of Science and Technology Qingdao P. R. China

**Keywords:** co‐catalyze, laccase, lipase, mitomycin‐like compound, response surface methodology

## Abstract

To reach the excellent yield as well as environmental friendliness, an efficient one‐pot process for the synthesis of 2‐methyl‐3‐*n‐*butylaminoyl‐1,4‐benzoquinone, a mitomycin‐like compound by the domino reaction of 2‐methyl‐1,4‐hydroquinone and butylamine using laccase/lipase as co‐catalysts, has been developed. In this present study, the process proposed here was further improved by optimizing the relevant factors using the response surface methodology based on Box–Benkhen Design. The optimum condition that afforded the highest yield (98%) of 2‐methyl‐3‐*n‐*butylaminoyl‐1,4‐benzoquinone was obtained as follows: molar ratio of amines to hydroquinones 1.16:1, activity ratio of laccase to lipase 1.14:2, and reaction temperature 38.9°C. The results obtained indicate that this process may be useful as a green alternative method for higher yield production of mitomycin analogs.

**Abbreviations**
ANOVA: analysis of variance
BBD: Box Behnken design
MMC: mitomycin C
RSM: response surface methodology

## 1 INTRODUCTION

Mitomycin C (MMC) is one of the most important members of mitomycins family that has been widely used as strong antineoplastic antibiotic for treating various tumors associated with gastric, pancreatic, colorectum, mammary, lung, etc. 1. The biological activity of mitomycin C is based on an aminoquinone moiety which is also prevalent in other antineoplastic drugs such as actinomycin and streptonigrin 2. Its clinical application, however, to a large extent, was powerfully restricted due to its high toxicity resulted from the low anti‐tumor selectivity 3. Therefore, there is an urgent need for novel MMC derivatives and analogues in order to increase its therapeutic efficacy. Although MMC and its analogues have been synthesized by various chemical methods 4, 5, it still fails to reach the excellent yield as well as environmental friendliness due to the poor reaction selectivity and tedious reaction steps etc.

Enzymatic domino reactions have been paid considerable attention in the past decade, which offers two distinct advantages including good yield of the final product and an asymmetric fashion for easily obtaining nonracemic products both as a result of the highly chemo‐, regio‐ or stereoselectivity of enzyme catalysts 6. Laccase (EC1.10.3.2), a multicopper‐containing polyphenol oxidase, can be applied for oxidizing phenolic and non‐phenolic compounds by converting them to the related reactive quinones 7. Laccase has attracted increasing interest in organic synthesis owing to its remarkable advantages including high stability in solution, mild reaction conditions, and good selectivity for phenolic substructures 8, 9, 10, 11. Laccase‐mediated amination is an efficient method for generating biologically active products, such as aminonaphthoquinone or aminoquinone derivatives. Niedermeyer et al. first found that fungal laccases from *Trametes* species and *Myceliophthora thermophila* can catalyze the synthesis of aminoquinones by the nuclear amination of *p*‐hydroquinones with primary aromatic amines 12. A fungal laccase from *Trametesvillosa* was successfully used to mediate the oxidation and cross‐coupling of two para‐dihydroxylated benzoic acid derivatives with 4‐aminobenzoic acid to synthesize aminobenzoquinones derivatives 13. Recently, Herter et al. 14 also reported the heteromolecular coupling of 2‐methoxy‐3‐methylhydroquinone with aliphatic and cyclic primary amines to form mitomycin‐like products mediated by *M. thermophila* laccase (MtL) and *P. cinnabarinus* laccase (PcL), respectively. Whereas this method gives a lower product yield that is less than 35% after 6 h of reaction. Moreover, the product formation in above mentioned laccase‐catalyzed reactions was influenced by many factors including the source of laccase, the electrophilicity of the hydroquinones, the nucleophilicity of the amines, ratio of amines to hydroquinones, temperature, and solubility, etc. 14, 15. Lipase, a promiscuous biocatalyst, has shown good catalytic abilities in C–C bond formation, C–heteroatom bond formation (e.g. C–N, C–O, C–S coupling), oxidative processes, and novel hydrolytic reactions 16, 17. Therefore, utilization of lipases as the biocatalysts for producing aminoquinone derivative by C–N coupling reaction between laccase generated quinones and amines may prove useful. A laccase‐lipase co‐catalytic system was used to catalyze the domino reaction between catechols and nucleophilic reagents including 1,3‐dicarbonyl compounds and aromatic amines and led to a remarkable increasing of the yield of the final products comparing with the reaction in the presence of laccase alone 18.

**SCHEME 1:**
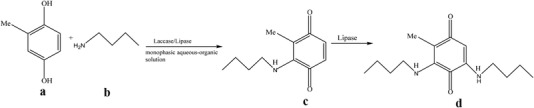
The synthesis of 2‐methyl‐3‐n‐butylaminoyl‐1,4‐benzoquinone mitomycin analogs co‐catalyzed by laccase/lipase through oxidation/amination reaction of 2‐methyl‐1,4‐hydroquinone with *n*‐butylamine

In this context, it is appropriate to mention here that the optimized conditions for enhancing the ability of laccase/lipase in catalyzing high yield production of aminoquinone derivatives by domino reaction has not been reported in literature so far. Herein, we investigated the feasibility of a cascade enzymatic synthesis of aminoquinone derivative, 2‐methyl‐3‐*n‐*butylaminoyl‐1,4‐benzo‐quinone, using 2‐methyl‐1,4‐hydroquinone and *n‐*butylamine as substrates co‐catalyzed by *Pleurotus ostreatus* laccase and *Trichosporon laibachii* lipase in a one‐pot process. The general reaction for the laccase/lipase catalyzed synthesis of 2‐methyl‐3‐*n‐*butylaminoyl‐1,4‐benzoquinone is shown in Scheme 1. In this work, the influence of important variables including the mole ratio of amines to hydroquinones (MR), activity ratio of laccase to lipase (AR), and reaction temperature (T) were investigated and optimized using the response surface methodology (RSM), employing a three‐variable, three‐level Box Behnken design (BBD) 19, 20. The optimum reaction conditions as well as the relationships between the factors and the responses (yield of 2‐methyl‐3‐*n‐*butylaminoyl‐1, 4‐benzoquinone) by RSM analysis were obtained.

**PRACTICAL APPLICATION**

Mitomycin C is one of the most important members of mitomycins family with extensive application in the treatment of various tumors associated with gastric, pancreatic, colorectum, mammary, lung, etc. Chemical methods for synthesis of Mitomycin C and its analogues still fail to reach the excellent yield as well as environmental friendliness due to the poor reaction selectivity and tedious reaction steps, etc. This paper presents an efficient one‐pot process for the synthesis of 2‐methyl‐3‐*n‐*butylaminoyl‐1, 4‐benzoquinone, a mitomycin‐like compound using laccase/lipase as co‐catalysts. Under the optimum condition, the highest yield of 98% was achieved. This process may be useful as a green alternative method for higher yield production of mitomycin analogs.

## 2 MATERIALS AND METHODS

### 2.1 Enzymes and chemicals

An immobilized lipase from *T. laibacchii* CBS5791 was used as catalyst in combination with laccase from *P. ostreatus* for oxidation/amination reaction of 2‐methyl‐1,4‐hydroquinone with *n‐*butylamine. The immobilized *T. laibacchii* lipase was prepared using an in situ immobilization method as described in our previous work in which an aqueous two‐phase system with 12% PEG 4000/13% K_2_HPO_4_ was used to purify the lipase, diatomites was used as a supporter, and glutaraldehyde was used as a cross‐linker 21. Laccase from *P. ostreatus* was purified and immobilized on glass beads by using the methods reported by Macellaro et al. 22. The immobilized *P. ostreatus* laccase was prepared in 50 mM NaP buffer, pH 6.5 at room temperature for 1 h. 2‐Methyl‐1,4‐hydroquinone (**a**, 98%) and *n‐*butylamine (**b**, 99.7%) were both purchased from Shanghai Aladdin Biochemical Technology and used as received without further purification.

### 2.2 Lipase/laccase co‐catalyzed oxidation/amination reaction of 2‐methyl‐1, 4‐hydroquinone with *n‐*butylamine

The target product 2‐methyl‐3‐*n‐*butylaminoyl‐1,4‐benzoquinone (**c**) was synthesized via the oxidation reaction of 2‐methyl‐1,4‐hydroquinone (**a**) followed by amination of the formed intermediate 2‐methyl‐1,4‐benzoquinone with *n‐*butylamine (**b**) in a 20‐mL reaction vessel. The reactions were performed using the immobilized *T. laibacchii* lipase in conjugation with immobilized *P. ostreatus* laccase pH 7. Methanol (2–4%, v/v) was used as a co‐solvent to improve the dissolution of the substrates. The reaction mixture (10 mL) contained 25–50 U/mL of each biocatalyst. The reactions were carried out at 40–50°C and shaken at 200 rpm in a water bath shaker for 20–24 h. Samples were withdrawn at specific time intervals from reaction mass and analyzed by HPLC. All reactions were carried out in duplicate.

The immobilized lipase was removed by filtration and the supernatant solution was extracted three times with ethyl acetate. The ethyl acetate layer was concentrated by being dried over MgSO_4_ and finally evaporated under reduced pressure. Then the concentrated organic layer was applied to a Strata™ SDB‐L column with styrene‐divinylbenzene polymer as absorbent material (1000 mg, 6 ml, Phenomennex, USA) for SPE. The reaction product **c** was eluted with methanol/0.5% aqueous acetic acid (80/20, v/v). The by‐products and residual substrate were eluted with methanol/deionized water (65:35, v/v). The pure methanol was used to elute the diaminated products **d**. The methanol of samples was removed by rotary evaporation at 30°C.

### 2.3 HPLC analysis

The samples were withdrawn at various intervals and were analyzed with an Agilent 1200 series device equipped with a G1312BB inpump SL pump, G1367LHip‐ALS SL auto sampler, and a G1315A DAD detector (Agilent Technologies, Santa Clara, USA). The column employed was an Agilent ZORBAX Eclipse XDB‐C18 column (250 × 4.6 mm, 5 µm). The mobile phase consisted of a gradient of 60–100% methanol in water within 12 min at a flow rate of 0.5 mL/min and the sample injection volume was 5 µL. The column was maintained at 30°C and the detection wave length was 227 nm. The retention times of 2‐methyl‐3‐*n‐*butylaminoyl‐1,4‐benzoquinone (**c**) and 2‐methyl‐3,5‐*n‐*butylaminoyls‐1,4‐benzoquinone (**d**) were 4.13 and 4.74 min, respectively.

### 2.4 Experimental design and statistical analysis

Three factors including the mole ratio of amines to hydroquinones (MR), activity ratio of laccase to lipase (AR), and reaction temperature (T) were selected as independent variables and the yield of product **c** was chosen as response variable. Table 1 shows the independent variables, their levels, and real values. The optimum conditions for synthesis of product **c** by lipase/laccaseco‐catalyzed were investigated based on a three‐level, three‐variable Box–Behnken design by using the Design Expert software (version 8.0.7, Stat‐Ease, Minneapolis, USA). The experiment design and the yield results are presented in Table 3. A quadratic polynomial equation as given in Equation (1) was developed to study the effects of variables on the yield of product **c**.

(1) $$Y=A_{0}+\sum_{i=1}^{n}A_{i}X_{i}+\sum_{i=1}^{n}A_{ii}{X_{i}}^{2}+\sum_{i=1}^{n-1}\sum_{j=i+1}^{n}A_{ij}X_{i}X_{j}$$

where *Y* is the predicted response; *A*_0_ is the constant; and *A_i_*, *A_ii_*, and *A_ij_* are the regression coefficients estimated by the multiple regressions for the linear, quadratic, and cross‐product effects of the factors, respectively; *X_i_* and *X_j_* are the independent variables. The coefficient of determination (*R* ^2^) and the adjusted coefficient of determination (*R* ^2^_Adj_) were used to check the fit of regression model. A regression analysis and the plotting of response surface were performed to fit quadratic polynomial equations for all response variables and obtain the optimum conditions. Significance of data was tested using the analysis of variance.

**Table 1:** Factors and levels value used in the experimental design

		Levels		
Factors	Code	‐1	0	1
Mole ratio of amines to hydroquinones (MR)	*X_1_*	3:4	1:1	5:4
Activity ratio of laccase to lipase (AR)	*X_2_*	1:4	1:2	3:4
Reaction temperature (℃) (T)	*X_3_*	30	40	50

## 3 RESULTS AND DISCUSSION

### 3.1 Optimizing lipase/laccase co‐catalyzed synthesis of 2‐methyl‐3‐*n‐*butylaminoyl‐1, 4‐benzoquinone: RSM experiments and model fitting

RSM is a widely used statistical tool that is useful for modeling and analysis of problems in which a response of interest is influenced by various parameters. It helps to establish the optimum conditions with a minimum number of experiments, and also to analyze the mutual interaction between the independent variables 23, 24. The best fitting model was established based on the Box–Behnken Design and results of the experiments by a multiple regression analysis using the Design Expert 8.0.7 software. The experimental results presented in Table 2 were subjected to regression analysis to evaluate the effects of mole ratio of amines to hydroquinones (*X*_1_), activity ratio of laccase to lipase (*X*_2_), and reaction temperature (*X*_3_) on the yield of product **c** (*Y*). The terms found to be significant were combined into the following fitted quadratic polynomial equation (Equation (2)):

(2) $$\begin{array}{ccc} Y & = & 85.69+8.68X_{1}+12.19X_{2}+4.66X_{3}+5.91X_{1}X_{2}\\ & & -\;16.49X_{1}X_{3}+8.71X_{2}X_{3}-11.21{X_{1}}^{2}-33.34{X_{2}}^{2}\\ & & -\;19.72{X_{3}}^{2} \end{array}$$

**Table 2:** Box–Behnken design and results of response surface methodology for yield of product **c**

	Variables			
Exp. No.	*X_1_*	*X_2_*	*X_3_*	Response Yield^a^ of product **c** (%)
1	−1	−1	0	28.16
2	−1	1	0	38.81
3	1	−1	0	31.67
4	1	1	0	65.95
5	0	−1	−1	21.61
6	0	−1	1	17.38
7	0	1	−1	30.47
8	0	1	1	61.09
9	−1	0	−1	25.85
10	1	0	−1	78.23
11	−1	0	1	64.28
12	1	0	1	50.71
13	0	0	0	87.09
14	0	0	0	86.42
15	0	0	0	83.57

a The yield of product c was counted based on a 100% conversion, estimated by HPLC‐analysis.

The oxidation/amination reaction of 2‐methyl‐1, 4‐hydroquinone with *n‐*butylamine co‐catalyzed by lipase/laccase in producing 2‐methyl‐3‐*n‐*butylaminoyl‐1,4‐benzoquinone is appropriately described with Equation (2). From Equation (2), it can be seen that all the linear terms and the terms that corresponded to a mutual interaction between *X*_1_ and *X*_2_ as well as *X*_2_ and*X*_3_ carried positive coefficients. That indicated these factors have a direct positive effect on the yield of product **c**. In contrast, all the quadratic terms (*X*_1_^2^, *X*_2_^2^, and *X*_3_^2^) and the interaction terms (*X*_1_ *X*_3_) had a negative effect on the yield of product **c**.

The results of analysis of variance and regression analysis of the fitted quadratic regression model are shown in Table 3. As seen from Table 3, the determination coefficient *R* ^2^ of 0.9941 indicates that 99.41% of the total sample variation for the yield of product **c** can be explained by the model. The present *R* ^2^ value reflects that the predicted values obtained by model fitting sufficiently correlate with the observed values. The adjusted *R* ^2^ value that is higher than 0.98 also indicates a very good fit between the observed and predicted responses. Larger the *F*‐value is, more significant the corresponding coefficient would be, by contrast, a very small *p*‐value (<0.0001) would indicate the significance of the corresponding coefficient. In this study, the *F*‐ratio of quadratic regression model was 94.05 with a low probability value (*p* < 0.0001), which means the model is well accurate for predicting the yield of product **c**. It can be observed from analysis of variance (Table 3) and Equation (2) that all the linear effects, all the quadratic effects, and the interaction effects (*X*_1_ *X*_3_ and *X*_2_ *X*_3_) had significant impact on the yield of product **c** (*p *< 0.001), while the model term of *X*_1_ *X*_2_ was significant at 5% level (*p *< 0.05). The variables with the largest effect on the yield of product **c** were the linear term of *X*_2_ and the quadratic term of *X*_2_ according to the *F*‐value. It was that the activity ratio of laccase to lipase (*X*_2_) being the key variable that afforded the highest impact on the formation of product c. The insignificant Lack‐of‐Fit *F*‐value of 4.35 further proves that the data in the experimental domain are well represented by the model and thus the model equation used is sufficient for predicting the yield of product **c** under any combination of values of the variables. Therefore, it is reasonable to use the quadratic polynomial model generated to explain the actual relationship between the response and the variables.

**Table 3:** Results of regression analysis and analysis of variance for the fitted quadratic polynomial models

	Yield of product **c**		
Source	Coefficient	*F*‐Value	*p*‐Value
Intercept	85.69		
*X_1_*	8.68	57.37	0.0006**
*X_2_*	12.19	113.04	0.0001**
*X_3_*	4.66	16.54	0.0097**
*X_1_X_1_*	−11.21	44.12	0.0012**
*X_1_X_2_*	5.91	13.28	0.0148*
*X_1_X_3_*	−16.49	103.44	0.0002**
*X_2_X_2_*	−33.34	390.38	<0.0001**
*X_2_X_3_*	8.71	28.88	0.0030**
*X_3_X_3_*	−19.72	136.56	<0.0001**

Source	Sums of squares	*df*	Mean square	*F*‐Value	*p*‐Value
Model	8897.89	9	988.65	94.05	<0.0001**
Residual	52.56	5	10.51		
Lack of fit	45.57	3	15.19	4.35	0.1926
Pure Error	6.99	2	3.49		
Cor Total	8950.45	14			
*R* ^2^ 0.9941	*R* ^2^_Adj_ 0.983	*R* ^2^_Pred_ 0.9168			

^*^ Significant at *p *< 0.05.

^**^ Significant at *p *< 0.01.

### 3.2 Interactive effects of different factors on the lipase/laccase co‐catalyzed oxidation/amination reaction of 2‐methyl‐1,4‐hydroquinone with *n‐*butylamine

**FIGURE 1:**
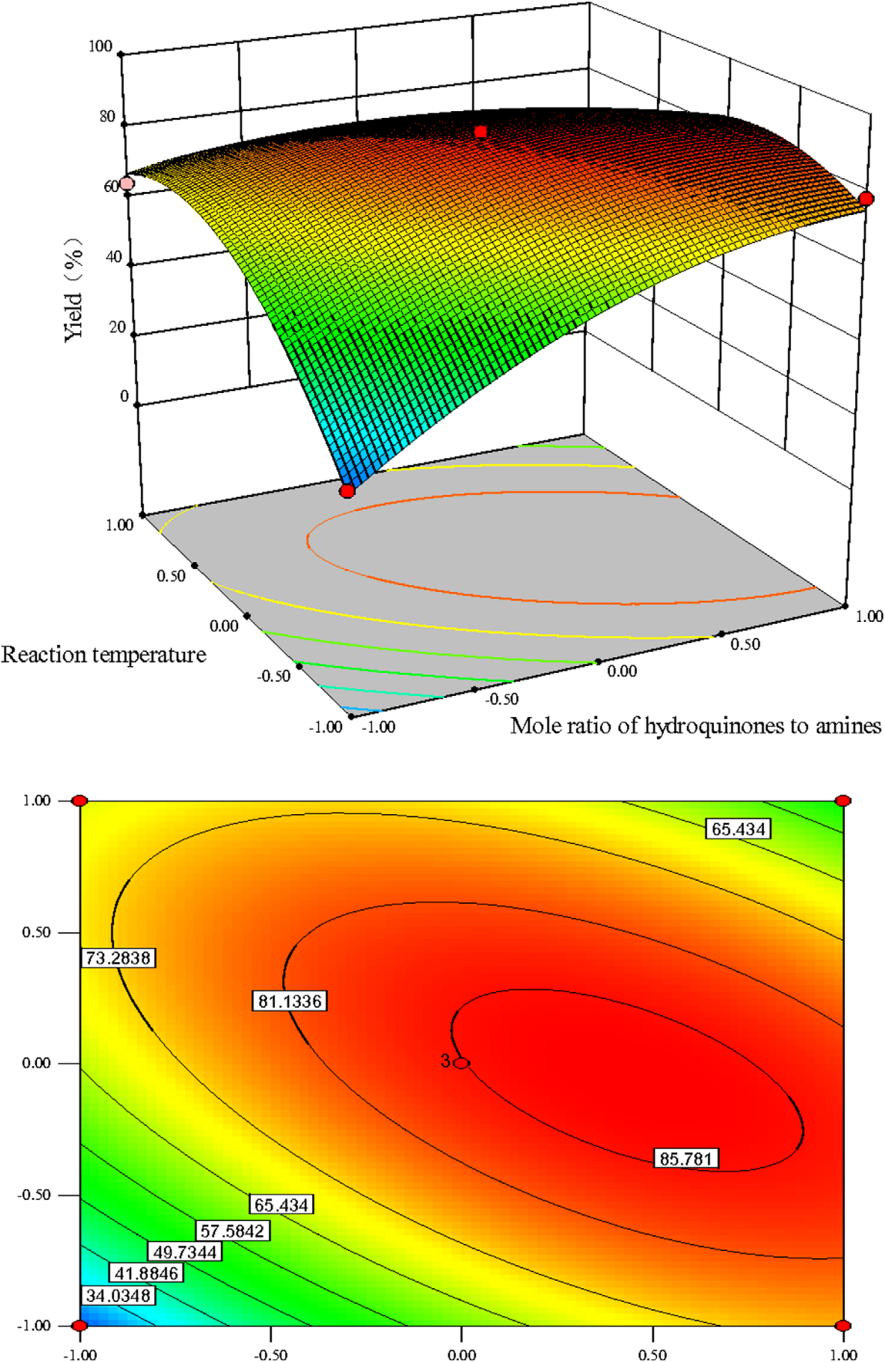
Response surface plots and contour plots for the effect of mole ratio of hydroquinones to amines (X1)/reaction temperature (X3) on the yield of product c with constant activity ratio of laccase to lipase (1:2)

**FIGURE 2:**
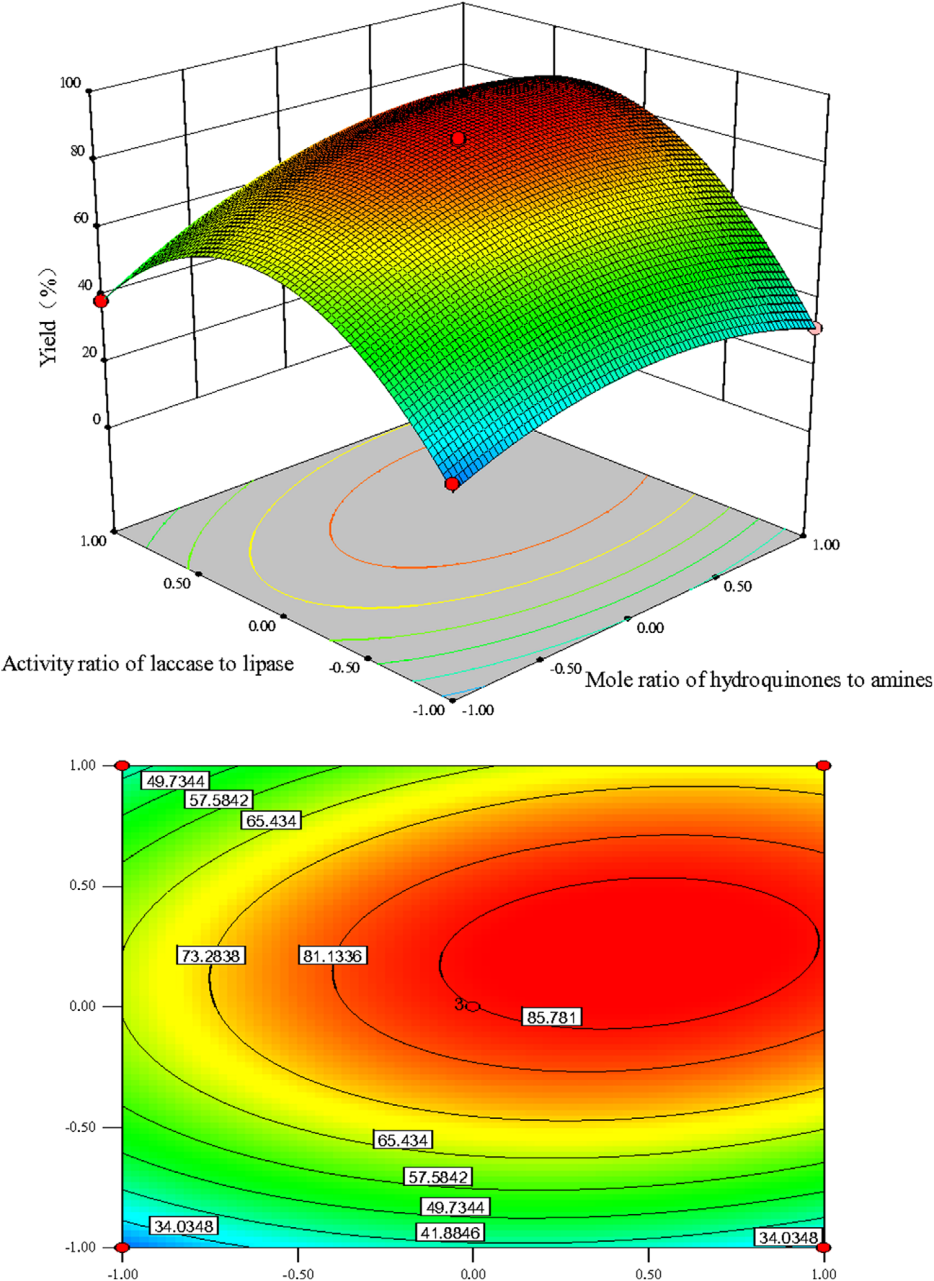
Response surface plots and contour plots for the effect of mole ratio of hydroquinones to amines (X1)/activity ratio of laccase to lipase (X2) on the yield of product c with constant reaction temperature (40℃)

The interactive effects of different factors including mole ratio of amines to hydroquinones (*X*_1_), activity ratio of laccase to lipase (*X*_2_) and reaction temperature (*X*_3_) on oxidation/amination reaction of 2‐methyl‐1,4‐hydroquinone with *n‐*butylamine catalyzed by lipase/laccase were investigated based on contour plots. According to Equation (2), it was obvious that mutual interactions between *X*_1_ and *X*_2_ as well as *X*_2_ and *X*_3_ with positive coefficients were synergistic, while the mutual interaction between *X*_1_ and *X*_3_ with negative coefficients was antagonistic. Among these interaction terms, the interaction effect of *X*_1_ *X*_3_ with F‐value of 103.44 in comparison with that of *X*_1_ *X*_2_ and *X*_2_ *X*_3_ has the highest impact on the yield of product **c**. Figure 1 illustrates the effects of varying mole ratio of hydroquinones: amines and temperature on the lipase/laccase co‐catalyzed production of product **c** at constant activity ratio of laccase to lipase of 1:2. It was evident that the reaction was sensitive to minor alterations of the test variables *X*_1_, *X*_2,_ and *X*_3_. The variables with the largest effect on the yield of product **c** were the linear term of *X*_2_ and the quadratic term of *X*_2_ according to the *F*‐value. The effect of activity ratio of laccase to lipase (*F*‐value 113.04) on the yield of product **c** was more impacting than the mole ratio of amines to hydroquinones (*F*‐value 57.37) and reaction temperature (*F*‐value16.54) in this enzymatic catalysis reaction. Generally, an elliptical contour plot means the interactions between the corresponding variables are significant, while a circular contour plot indicates that the interactions between them are negligible. The contour plots of the response in Figure 1, 2, 3 are drawn as a function of two factors at a time, holding another factor at the zero level. It was clear that the mutual interaction between the independent variables, *X*_1_ and *X*_3_, exhibited a strong and perfect interaction as implied by the obtained elliptical contours with *F*‐value of 103.44 (Figure 1).

**FIGURE 3:**
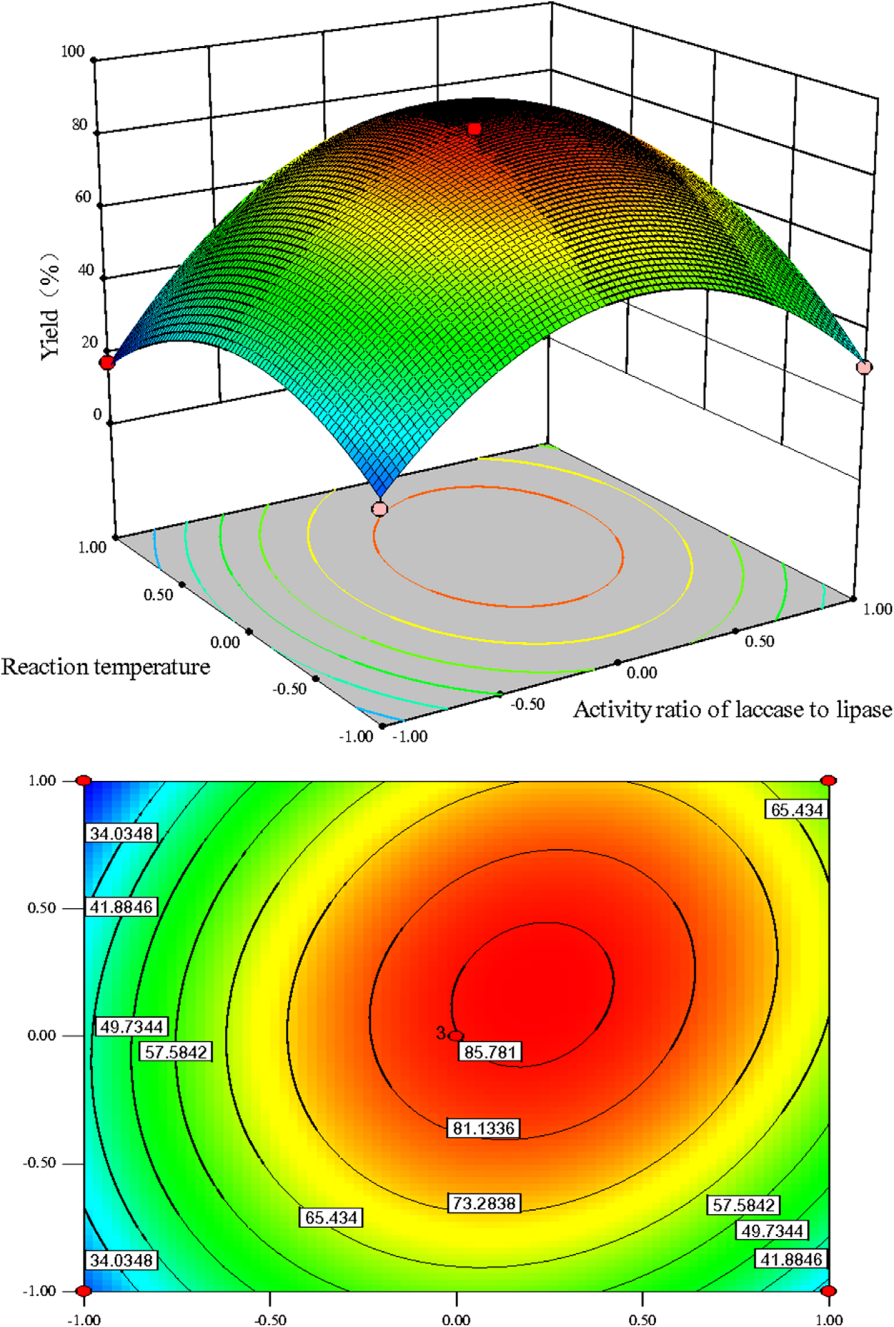
Response surface plots and contour plots for the effect of activity ratio of laccase to lipase (X2)/reaction temperature (X3) on the yield of product c with constant mole ratio of amines to hydroquinones (1:1)

The influence of activity ratio of laccase to lipase on the response at constant mole ratio of amines to hydroquinones and reaction temperature (zero level) are shown in Figures 2 and 3, respectively. The activity ratio of laccase to lipase had significant effect on the response. The effect of mole ratio of amines to hydroquinones on response at constant reaction temperature and activity ratio of laccase to lipase, are shown in Figures 1 and 2, respectively. The effect of reaction temperature on the yield of product **c** at constant activity ratio of laccase to lipase and mole ratio of amines to hydroquinones are shown in Figures 1 and 3, respectively. The interaction between mole ratio of amines to hydroquinones and reaction temperature (*p*‐value 0.0002) was statistically more significant than the interaction between reaction temperature and activity ratio of laccase to lipase (*p*‐value 0.003). However, the mutual interaction between mole ratio of amines to hydroquinones and activity ratio of laccase to lipase was less favorable (*F*‐value 13.28) when compared to these two interactions with *F*‐value of 103.44 and 28.88, respectively.

Generally, reaction temperature has important influence on the enzyme activity and stability which are considered to be the most important factors in enzymatic reactions. The yield of product **c** increased with temperature up to the optimum temperature, after which the degree of production achieved had a tendency to decrease (Figures 1 and 3). The effect of temperature on conversion in the case of enzymatic reactions was also well reported in many documents 25, 26. This effect may be attributed to the elevated kinetic energy within the system which would promote effective collisions between the enzyme and substrate molecule, resulting in the improvement in the production rate of the product 27, 28, 29. However, further increasing the temperature did not improve the yield of product **c**, possibly due to decreased stability and partial inactivation of the enzyme. Therefore, an appropriate reaction temperature for high yield is of great importance.

Lipases have been widely used in Michael reactions between different substrates such as active methylene compounds and chalcones 30, 4‐hydroxycoumarin and α, β‐unsaturated enones 31, primary or secondary amines, acrylonitrile, etc. 32. The synthesis of aminoquinones by laccase initiated oxidation of *p*‐hydroxyquinones followed by Michael addition catalyzed by lipase between the laccase‐generated o‐quinone and primary aromatic amines, has been well demonstrated 18. This co‐catalytic enzyme system of lipase in conjugation with laccase provided a higher rate of the reaction and higher yield of the product than the reaction without lipase. To the best of our knowledge, however, the effect of activity ratio of laccase to lipase in this co‐catalytic enzyme system has almost not reported.

Here, we found that laccase/lipase activity ratio of has the largest effect on the yield of product **c**. Different laccase/lipase activity ratio of resulted in clear differences in response (Table 3). At the optimum laccase/lipase activity ratio, the response of the yield of product **c** was evidently increased. However, poor response is also relative with inappropriate laccase/lipase activity ratio (Figure 1a and c). Therefore, the proportion of each enzyme is crucial for the efficiency of multienzymatic systems 33. The mechanism behind this effect was most likely that the inhibition caused by laccase‐generated *o*‐quinone can be reduced by adding a certain amount of lipase, while excessive lipase might result in the percentage of diaminated product **d**. The influence of enzymes activity ratio on the reaction outcome has also been investigated in enzymatic cascade reaction for the synthesis of imino sugar precursors 34 and oxidation of phenolic compounds 35. It is believed that the synergistic action of multienzyme can enhance the catalytic efficiency of the whole system 36. The ratio of each enzyme is critical factor affecting end‐product yield, when constructing multiple enzymes mediated cascade reactions 37.

In this study, we aimed to obtain the monoaminated product **c** in high yield by nuclear amination reaction between the laccase‐generated 2‐methyl‐1,4‐benzoquinone and *n‐*butylamine catalyzed by lipase. An increased formation of diaminated products with excessively applied amine donors has been already demonstrated 38, 39. Therefore, an optimum mole ratio of amines to hydroquinones is very important for obtaining high yield products. It can be seen from Figures 1 and 2 that a percentage of yield of product **c** more than 85% could be attained when the molar ratio of amines to hydroquinones was between 1:1 and 5:4. Hertera et al. 40 performed the synthesis of monoaminated mitomycin‐like products using laccases, and their results show that the monoaminated products were mainly obtained only at pH 7 and the ratio of benzoquinone to amine was 1:1–2. They found that an excessive amine with hydroquinones/amines molar ratio of 2:5 resulted in formation of the diaminated product with a HPLC‐yield of 78.8%.

### 3.3 Obtaining the optimum synthesis conditions and verification of the model

In the present work, the predicted percentage yield value was selected for optimizing the conditions for the lipase/laccase co‐catalyzed oxidation/amination reaction. The optimum conditions that afforded the highest percentage yield were obtained as follows: mole ratio of amines to hydroquinones 1.16:1, activity ratio of laccase to lipase 1.14:2, and reaction temperature 38.9°C.

Based on the established optimum conditions by RSM, a validation test with triplicate was carried out within the design space. The average yield value 87.17% obtained was observed under the optimum condition, which is very close to the predicted value (88.61%). This indicates that the model can be considered quite reliable for predicting the effect of variables including mole ratio of amines to hydroquinones, activity ratio of laccase to lipase, and reaction temperature on the yield of product.

## 4 CONCLUDING REMARKS

The present study successfully evaluated the optimized conditions suggested by the response surface methodology based on BBD utilizing laccase/lipase as co‐biocatalysts for producing mitomycin analogs (aminonaphthoquinones) via a one pot process. Considerably high yield of product **c** was observed under the optimum condition with molar ratio of amines to hydroquinones 1.16:1, activity ratio of laccase to lipase 1.14:2, and reaction temperature 38.9°C. A model based on RSM experimental results was developed that was verified as quite reliable for predicting the effects of significant factors on the response. The optimized conditions revealed by the BBD design may prove beneficial for higher yield of monoaminated mitomycin‐like products, an important aspect in the large scale production of mitomycin analogs. However, in order to have better understanding of one‐pot process for synthesis of aminonaphthoquinones catalyzed by co‐catalytic system of laccase/lipase, more researches on the synergistic catalytic mechanism of bienzyme are needed.

## CONFLICT OF INTEREST

The authors have declared no conflict of interest.
